# Dineodymium(III) ditungstate(VI), Nd_2_W_2_O_9_

**DOI:** 10.1107/S1600536808009914

**Published:** 2008-05-03

**Authors:** Peter Held, Petra Becker

**Affiliations:** a Institut für Kristallographie, Universität zu Köln, Zülpicher Strasse 49b, D-50674 Köln, Germany

## Abstract

Single crystals of monoclinic Nd_2_W_2_O_9_ were obtained by growth from tungsten borate flux in an atmosphere of air. The crystal structure consists of chains of distorted [WO_6_] octa­hedra that run along the *c* axis of the structure, and of [NdO_9_] polyhedra that are connected *via* common faces and common edges to form a three-dimensional framework.

## Related literature

For literature on related structures, see: Lacorre *et al.* (2000 ▶), Goutenoire *et al.* (2000 ▶) and Evans *et al.* (2005 ▶) for La_2_Mo_2_O_9_; Laligant *et al.* (2001 ▶) for La_2_W_2_O_9_; Yoshimura *et al.* (1976 ▶) for Ce_2_W_2_O_9_; Borisov & Klevtsova (1970 ▶) for Pr_2_W_2_O_9_; Aruga *et al.* (2005 ▶) for Eu_2_W_2_O_9_.

## Experimental

### 

#### Crystal data

Nd_2_W_2_O_9_
*M* *_r_* = 800.17
Monoclinic, 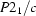

*a* = 7.6501 (11) Å
*b* = 9.8547 (10) Å
*c* = 9.2326 (13) Å
β = 107.538 (11)°
*V* = 663.69 (15) Å^3^
*Z* = 4
Mo *K*α radiation
μ = 49.96 mm^−1^
*T* = 290 (1) K
0.25 × 0.15 × 0.13 mm

#### Data collection

Stoe IPDSII diffractometer
Absorption correction: numerical [*X-SHAPE* (Stoe & Cie, 1999 ▶) and *X-RED* (Stoe & Cie, 2001 ▶)] *T*_min_ = 0.080, *T*_max_ = 0.469
15723 measured reflections
2330 independent reflections
2072 reflections with *I* > 2σ(*I*)
*R*_int_ = 0.088

#### Refinement

*R*[*F* ^2^ > 2σ(*F* ^2^)] = 0.037
*wR*(*F* ^2^) = 0.093
*S* = 1.09
2330 reflections
119 parameters
Δρ_max_ = 2.34 e Å^−3^
Δρ_min_ = −1.62 e Å^−3^

### 

Data collection: *X-AREA* (Stoe & Cie, 2001 ▶); cell refinement: *X-AREA*; data reduction: *X-AREA*; program(s) used to solve structure: *SIR92* (Altomare *et al.*, 1993 ▶); program(s) used to refine structure: *SHELXL97* (Sheldrick, 2008 ▶); molecular graphics: *ATOMS* (Dowty, 2002 ▶); software used to prepare material for publication: *SHELXL97*.

## Supplementary Material

Crystal structure: contains datablocks I, global. DOI: 10.1107/S1600536808009914/si2082sup1.cif

Structure factors: contains datablocks I. DOI: 10.1107/S1600536808009914/si2082Isup2.hkl

Additional supplementary materials:  crystallographic information; 3D view; checkCIF report

## Figures and Tables

**Fig. 1.**
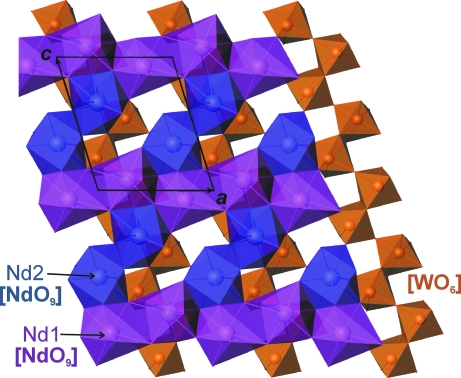
Projection along [010] of a fraction of the crystal structure of Nd2W2O9, showing one sheet of [NdO9] polyhedra that lies parallel to the a-c plane, and one layer of isolated chains of alternately edge- and corner-sharing [WO6] octahedra. Large purple and violet spheres denote Nd1 and Nd2 atoms, respectively, smaller orange spheres denote W atoms. Oxygen atoms are indicated by the corners of the coordination polyhedra and are not drawn.

**Fig. 2.**
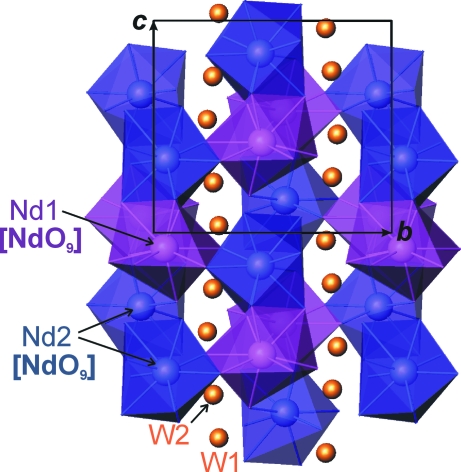
View of the structure of Nd2W2O9 along the a-axis, emphasizing the arrangement and mutual connection of [NdO9] polyhedra sheets that lie parallel to the a-c plane. Nd atoms are denoted by large purple and violet spheres. Tungsten atoms (marked with smaller orange spheres) occupy interstitial space between the sheets of Nd coordination polyhedra and are arranged in layers parallel to the a-c plane. Oxygen atoms are indicated by the corners of the coordination polyhedra and are not drawn.

**Fig. 3.**
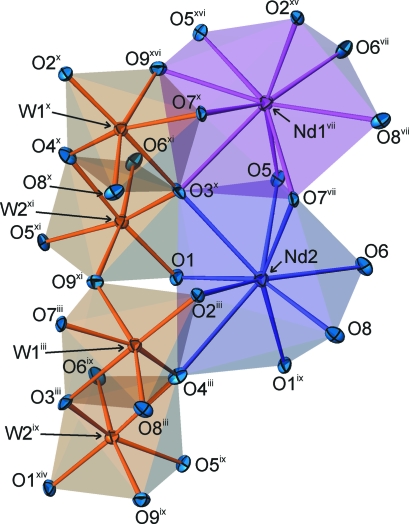
Fraction of the structure of Nd2W2O9 with atomic numbering scheme (projection approximately along the a-axis). Coordination polyhedra are indicated as a guide for the eye. Atoms are drawn as 50% probability ellipsoids. (Symmetry codes: (iii) -x + 1, -y + 1, -z; (vii) x, -y + 3/2, z + 1/2; (ix) -x, -y + 1, -z; (x) -x + 1, y - 1/2, -z + 1/2; (xi) -x, y - 1/2, -z + 1/2; (xiv) x, -y + 1/2, z - 1/2; (xv) x, y, z + 1; (xvi) -x, -y + 1, -z + 1).

## supplementary crystallographic information

### Comment

The crystal structure of the title compound consists of two symmetrically non-equivalent Nd atoms that both are ninefold coordinated by oxygen. Nd - O bond lengths range from 2.377 (6) Å to 3.096 (7) Å for Nd1 and from 2.447 (7) Å to 2.743 (8) Å for Nd2. (The distance to the next nearest cation, W1 in case of Nd1 with distance Nd1 - W1 = 3.3885 (7) Å, W2 in case of Nd2 with distance Nd2 - W2 = 3.3800 (7) Å, is taken as the limit of the first oxygen coordination surrounding of Nd). The [NdO_9_] polyhedra can be described as distorted capped square antiprisms for both Nd atoms. The polyhedra of Nd1 are sharing edges, thus forming chains that run along the *a*-axis of the structure (Fig. 1). Parallel to the *a-c* plane of the structure these chains are linked by dimers of edge-sharing coordination polyhedra of Nd2 (groups [Nd_2_O_16_]). The polyhedra dimers of Nd2 and the polyhedra chains of Nd1 are connected *via* common faces (*i.e.* three common oxygen ligands) between a Nd2 polyhedron and a Nd1 polyhedron, and a common edge of the Nd2 polyhedron and an adjacent Nd1 polyhedron (for the atomic numbering scheme see Fig. 3). From this linkage sheets of [NdO_9_] polyhedra parallel to the *a-c* plane result (Fig. 1). Along the *b*-axis the sheets are stacked in parallel with a translation of *c*/2, and are connected by common edges of Nd1 and Nd2 polyhedra alternatingly to neighbouring polyhedra sheets on both sides. This connection scheme results in a three-dimensional framework of [NdO_9_] polyhedra with narrow channels along the *c*-axis, where tungsten atoms are located. Within the [NdO_9_] polyhedra sheets Nd1 - Nd2 distances as short as 3.7787 (9) Å occur (face sharing of [NdO_9_] polyhedra).

Between the [NdO_9_] polyhedra sheets chains of distorted [WO_6_] octahedra are running along the *c*-axis (see Fig. 1 and Fig. 2). The octahedra chains consist of pairs of edge-sharing [WO_6_] units, each pair combining an octahedron [W1 O_6_] and an octahedron [W2 O_6_]. The octahedra pairs are connected *via* common corners O9 to infinite chains. W—O bonds to bridging oxygen atoms are elongated with bond lengths ranging from 1.855 (7) Å to 2.202 (7) Å. All oxygen atoms are simultaneously ligands of neodymium and of tungsten.

Borisov & Klevtsova (1970) published the structure of Pr_2_W_2_O_9_, however, with rather large uncertainty of the oxygen positions. Nd_2_W_2_O_9_ turns out to be isomorphous to this compound, but it should be noted that in Pr_2_W_2_O_9_ the coordination of one of the Pr atoms was regarded as eightfold, only, while the other is ninefold coordinated, as both Nd atoms are in Nd_2_W_2_O_9_. Due to this, in Pr_2_W_2_O_9_ the Pr coordination polyhedra are not connected to sheets but only to stripes parallel to the *a-c* plane of the structure. The inclusion of nine oxygen atoms to the coordination surrounding of Nd1 in Nd_2_W_2_O_9_ is meaningful, both, with respect to the connection scheme of Nd coordination polyhedra and regarding the Nd - O distances, where a distinct gap between distances of the nine oxygen atoms included in the coordination polyhedron and distances of further oxygen atoms is seen. All further oxygen atoms have distances Nd1 - O equal or larger than 3.4694 Å, which is more than the shortest Nd—W distance.

After the discovery of fast oxygen ion conduction in La_2_Mo_2_O_9_ by Lacorre *et al.* (2000) interest in *RE*_2_*M*_2_O_9_ (*RE* = rare earth element, *M* = Mo, W) compounds renewed. For La compounds La_2_*M*_2_O_9_ (*M* = Mo, W) evidence for the occurrence of a high-temperature (space group *P*2_1_3) and a low-temperature modification (space group *P*2_1_ for La_2_Mo_2_O_9_, space group *P*1 for La_2_W_2_O_9_), together with their structure determination was given by Goutenoire *et al.* (2000), Laligant *et al.* (2001) and Evans *et al.* (2005); a similar polymorphy had been already presumed earlier for Ce_2_W_2_O_9_ by Yoshimura *et al.* (1976). Recently, the compound Eu_2_W_2_O_9_ was mentioned to be isomorphous to Pr_2_W_2_O_9_ (and hence to Nd_2_W_2_O_9_) by Aruga *et al.* (2005).

### Experimental

Light purple prismatic single crystals of Nd_2_W_2_O_9_ were obtained by growth from tungsten borate flux using a melt of composition Nd_2_O_3_: B_2_O_3_: WO_3_ = 22.5: 25: 52.5. An appropriate homogenized powder mixture of Nd_2_O_3_ (99.9%, Alfa Aesar), B_2_O_3_ (99.98% Alfa Aesar) and WO_3_ (99.8%, Alfa Aesar) was heated in a covered platinum crucible in air atmosphere to 1423 K and subsequently cooled at a rate of 3 K h^-1^ to 1173 K. Transparent single crystals of the title compound were separated mechanically from the tungsten borate flux.

### Refinement

Refinement of F^2^ against ALL reflections. The weighted *R*-factor *wR* and goodness of fit S are based on F^2^, conventional *R*-factors *R* are based on F, with F set to zero for negative F^2^. The threshold expression of F^2^ > 2sigma(F^2^) is used only for calculating *R*-factors(gt) *etc*. and is not relevant to the choice of reflections for refinement. *R*-factors based on F^2^ are statistically about twice as large as those based on F, and R– factors based on ALL data will be even larger.

### Crystal data

**Table:**

Nd_2_W_2_O_9_	*F*_000_ = 1360
*M**_r_* = 800.17	*D*_x_ = 8.008 Mg m^−^^3^
Monoclinic, *P*2_1_/*c*	Mo *K*α radiation λ = 0.71073 Å
*a* = 7.6501 (11) Å	Cell parameters from 25 reflections
*b* = 9.8547 (10) Å	θ = 20.1–27.6º
*c* = 9.2326 (13) Å	µ = 49.96 mm^−^^1^
β = 107.538 (11)º	*T* = 290 (1) K
*V* = 663.69 (15) Å^3^	Prism, light purple
*Z* = 4	0.25 × 0.15 × 0.13 mm

### Data collection

**Table:**

Stoe IPDSII diffractometer	2330 independent reflections
Radiation source: fine-focus sealed tube	2072 reflections with *I* > 2σ(*I*)
Monochromator: graphite	*R*_int_ = 0.088
*T* = 290(1) K	θ_max_ = 32.2º
ω and φ scans	θ_min_ = 2.8º
Absorption correction: numerical[X-SHAPE (Stoe & Cie, 1999) and X-RED (Stoe & Cie, 2001)]	*h* = −11→11
*T*_min_ = 0.080, *T*_max_ = 0.469	*k* = −14→14
15723 measured reflections	*l* = −13→13

### Refinement

**Table:**

Refinement on *F*^2^	Secondary atom site location: difference Fourier map
Least-squares matrix: full	*w* = 1/[σ^2^(*F*_o_^2^) + (0.0439*P*)^2^ + 16.1611*P*] where *P* = (*F*_o_^2^ + 2*F*_c_^2^)/3
*R*[*F*^2^ > 2σ(*F*^2^)] = 0.037	(Δ/σ)_max_ < 0.001
*wR*(*F*^2^) = 0.093	Δρ_max_ = 2.34 e Å^−^^3^
*S* = 1.09	Δρ_min_ = −1.62 e Å^−^^3^
2330 reflections	Extinction correction: SHELXL97 (Sheldrick, 2008), Fc^*^=kFc[1+0.001xFc^2^λ^3^/sin(2θ)]^-1/4^
119 parameters	Extinction coefficient: 0.00391 (19)
Primary atom site location: structure-invariant direct methods	

### Special details

**Table:**

Experimental. A suitable single-crystal was carefully selected under a polarizing microscope and mounted in a glass capillary. The scattering intensities were collected on an imaging plate diffractometer (*IPDS* II, Stoe & Cie) equipped with a fine focus sealed tube X-ray source (Mo Kα, λ = 0.71073 Å) operating at 50 kV and 30 mA. Intensity data for the title compound were collected at room temperature by ω-scans in 180 frames (0 < ω < 180°; φ = 0° and 90°, Δω = 2°, exposure time of 10 min) in the 2Θ range 2.29 to 59.53°. Structure solution and refinement were carried out using the programs *SIR92* (Altomare *et al.*, 1993) and *SHELXL97* (Sheldrick, 2008). A numerical absorption correction (*X-RED* (Stoe & Cie, 2001) was applied after optimization of the crystal shape (*X-SHAPE* (Stoe & Cie, 1999)). The last cycles of refinement included atomic positions and anisotropic parameters for all atoms. The final difference maps were free of any chemically significant features. The refinement was based on F^2^ for ALL reflections.
Geometry. All e.s.d.'s (except the e.s.d. in the dihedral angle between two l.s. planes) are estimated using the full covariance matrix. The cell e.s.d.'s are taken into account individually in the estimation of e.s.d.'s in distances, angles and torsion angles; correlations between e.s.d.'s in cell parameters are only used when they are defined by crystal symmetry. An approximate (isotropic) treatment of cell e.s.d.'s is used for estimating e.s.d.'s involving l.s. planes.

### Fractional atomic coordinates and isotropic or equivalent isotropic displacement parameters (Å^2^)

**Table:**

	*x*	*y*	*z*	*U*_iso_*/*U*_eq_	
W1	0.57359 (5)	0.72579 (4)	−0.03462 (4)	0.01073 (11)	
W2	−0.07053 (5)	0.75136 (4)	0.26320 (4)	0.01058 (11)	
Nd1	0.28098 (7)	0.95544 (5)	0.07401 (5)	0.01298 (13)	
Nd2	0.22931 (7)	0.55245 (5)	0.15396 (5)	0.01243 (13)	
O1	−0.0113 (10)	0.3795 (7)	0.0941 (7)	0.0131 (12)	
O2	0.4920 (10)	0.5969 (7)	−0.1761 (7)	0.0144 (12)	
O3	0.7367 (9)	0.8644 (7)	0.1417 (7)	0.0128 (12)	
O4	0.7687 (10)	0.6210 (8)	0.0779 (8)	0.0152 (13)	
O5	0.0438 (10)	0.5887 (7)	0.3447 (8)	0.0139 (12)	
O6	0.0995 (10)	0.7810 (7)	0.1630 (8)	0.0149 (13)	
O7	0.4449 (9)	0.8935 (6)	−0.1077 (7)	0.0109 (11)	
O8	0.4091 (10)	0.7091 (8)	0.0739 (8)	0.0147 (12)	
O9	−0.2605 (10)	0.6904 (8)	0.3610 (7)	0.0143 (13)	

### Atomic displacement parameters (Å^2^)

**Table:**

	*U*^11^	*U*^22^	*U*^33^	*U*^12^	*U*^13^	*U*^23^
W1	0.01099 (18)	0.01014 (18)	0.01053 (17)	0.00011 (11)	0.00245 (12)	0.00011 (11)
W2	0.01064 (18)	0.01036 (17)	0.01018 (17)	0.00025 (11)	0.00230 (12)	0.00037 (11)
Nd1	0.0138 (2)	0.0122 (2)	0.0118 (2)	−0.00143 (15)	0.00216 (16)	0.00027 (15)
Nd2	0.0124 (2)	0.0116 (2)	0.0123 (2)	−0.00062 (15)	0.00227 (16)	0.00016 (15)
O1	0.016 (3)	0.009 (3)	0.011 (3)	0.002 (2)	−0.001 (2)	0.003 (2)
O2	0.022 (3)	0.010 (3)	0.009 (3)	−0.002 (2)	0.001 (2)	−0.003 (2)
O3	0.015 (3)	0.011 (3)	0.011 (3)	0.006 (2)	0.001 (2)	0.001 (2)
O4	0.013 (3)	0.017 (3)	0.012 (3)	0.006 (2)	−0.001 (2)	0.005 (2)
O5	0.018 (3)	0.009 (3)	0.012 (3)	0.002 (2)	0.001 (2)	0.004 (2)
O6	0.013 (3)	0.015 (3)	0.019 (3)	−0.004 (2)	0.008 (2)	0.004 (2)
O7	0.014 (3)	0.006 (3)	0.011 (3)	0.002 (2)	0.001 (2)	0.002 (2)
O8	0.011 (3)	0.019 (3)	0.016 (3)	−0.004 (2)	0.007 (2)	−0.001 (3)
O9	0.017 (3)	0.015 (3)	0.012 (3)	0.001 (2)	0.007 (2)	0.002 (2)

### Geometric parameters (Å, °)

**Table:**

W1—O2	1.794 (7)	Nd1—Nd2^vi^	3.7787 (9)
W1—O8	1.837 (7)	Nd1—Nd2^viii^	3.9479 (9)
W1—O4	1.855 (7)	Nd2—O8	2.331 (7)
W1—O7	1.938 (6)	Nd2—O7^vii^	2.379 (6)
W1—O9^i^	1.990 (7)	Nd2—O1	2.447 (7)
W1—O3	2.202 (7)	Nd2—O6	2.473 (8)
W1—Nd1^ii^	3.3885 (7)	Nd2—O1^ix^	2.485 (6)
W1—Nd2^iii^	3.4651 (7)	Nd2—O2^iii^	2.548 (8)
W1—Nd1	3.5344 (7)	Nd2—O5	2.598 (7)
W2—O1^iv^	1.796 (6)	Nd2—O3^x^	2.601 (7)
W2—O6	1.833 (7)	Nd2—O4^iii^	2.743 (8)
W2—O5	1.872 (6)	Nd2—W2^xi^	3.3800 (7)
W2—O3^v^	1.917 (6)	Nd2—W1^iii^	3.4651 (7)
W2—O9	2.020 (7)	O1—W2^xi^	1.796 (6)
W2—O4^v^	2.196 (7)	O1—Nd2^ix^	2.485 (6)
W2—Nd2^iv^	3.3800 (7)	O2—Nd1^vi^	2.439 (7)
W2—Nd2	3.3934 (7)	O2—Nd2^iii^	2.548 (8)
Nd1—O5^vi^	2.377 (6)	O3—W2^xii^	1.917 (6)
Nd1—O9^iv^	2.409 (8)	O3—Nd2^viii^	2.601 (7)
Nd1—O2^vii^	2.439 (7)	O3—Nd1^ii^	2.641 (7)
Nd1—O7	2.455 (7)	O4—W2^xii^	2.196 (7)
Nd1—O6	2.499 (7)	O4—Nd2^iii^	2.743 (8)
Nd1—O7^ii^	2.513 (7)	O5—Nd1^vii^	2.377 (6)
Nd1—O8	2.618 (8)	O7—Nd2^vi^	2.379 (6)
Nd1—O3^ii^	2.641 (7)	O7—Nd1^ii^	2.513 (7)
Nd1—O5^iv^	3.096 (7)	O9—W1^xiii^	1.990 (7)
Nd1—W1^ii^	3.3885 (7)	O9—Nd1^xi^	2.409 (8)
			
O2—W1—O8	100.9 (3)	O8—Nd1—Nd2^vi^	84.50 (16)
O2—W1—O4	93.3 (3)	O3^ii^—Nd1—Nd2^vi^	43.46 (15)
O8—W1—O4	102.3 (3)	W1^ii^—Nd1—Nd2^vi^	81.091 (15)
O2—W1—O7	108.8 (3)	W1—Nd1—Nd2^vi^	64.843 (14)
O8—W1—O7	84.6 (3)	O5^vi^—Nd1—Nd2^viii^	159.13 (18)
O4—W1—O7	155.3 (3)	O9^iv^—Nd1—Nd2^viii^	74.28 (17)
O2—W1—O9^i^	94.2 (3)	O2^vii^—Nd1—Nd2^viii^	38.63 (18)
O8—W1—O9^i^	160.6 (3)	O7—Nd1—Nd2^viii^	84.99 (15)
O4—W1—O9^i^	88.8 (3)	O6—Nd1—Nd2^viii^	118.17 (17)
O7—W1—O9^i^	79.0 (3)	O7^ii^—Nd1—Nd2^viii^	35.06 (14)
O2—W1—O3	166.6 (3)	O8—Nd1—Nd2^viii^	86.62 (16)
O8—W1—O3	88.9 (3)	O3^ii^—Nd1—Nd2^viii^	98.06 (15)
O4—W1—O3	75.5 (3)	W1^ii^—Nd1—Nd2^viii^	64.182 (13)
O7—W1—O3	81.0 (3)	W1—Nd1—Nd2^viii^	76.989 (16)
O9^i^—W1—O3	78.4 (3)	Nd2^vi^—Nd1—Nd2^viii^	116.337 (18)
O2—W1—Nd1^ii^	129.2 (2)	O8—Nd2—O7^vii^	80.4 (2)
O8—W1—Nd1^ii^	116.3 (2)	O8—Nd2—O1	149.9 (2)
O4—W1—Nd1^ii^	110.0 (2)	O7^vii^—Nd2—O1	129.0 (2)
O7—W1—Nd1^ii^	47.2 (2)	O8—Nd2—O6	71.9 (3)
O9^i^—W1—Nd1^ii^	44.4 (2)	O7^vii^—Nd2—O6	86.5 (2)
O3—W1—Nd1^ii^	51.18 (18)	O1—Nd2—O6	111.1 (2)
O2—W1—Nd2^iii^	45.4 (2)	O8—Nd2—O1^ix^	79.9 (2)
O8—W1—Nd2^iii^	122.5 (2)	O7^vii^—Nd2—O1^ix^	151.4 (2)
O4—W1—Nd2^iii^	51.9 (2)	O1—Nd2—O1^ix^	74.3 (3)
O7—W1—Nd2^iii^	142.0 (2)	O6—Nd2—O1^ix^	67.7 (2)
O9^i^—W1—Nd2^iii^	76.8 (2)	O8—Nd2—O2^iii^	81.3 (3)
O3—W1—Nd2^iii^	121.46 (17)	O7^vii^—Nd2—O2^iii^	74.0 (2)
Nd1^ii^—W1—Nd2^iii^	120.734 (19)	O1—Nd2—O2^iii^	99.8 (2)
O2—W1—Nd1	123.3 (2)	O6—Nd2—O2^iii^	149.1 (2)
O8—W1—Nd1	46.0 (2)	O1^ix^—Nd2—O2^iii^	122.9 (2)
O4—W1—Nd1	132.0 (2)	O8—Nd2—O5	128.2 (2)
O7—W1—Nd1	41.7 (2)	O7^vii^—Nd2—O5	73.2 (2)
O9^i^—W1—Nd1	115.0 (2)	O1—Nd2—O5	73.8 (2)
O3—W1—Nd1	70.08 (18)	O6—Nd2—O5	62.9 (2)
Nd1^ii^—W1—Nd1	72.141 (17)	O1^ix^—Nd2—O5	103.6 (2)
Nd2^iii^—W1—Nd1	166.031 (17)	O2^iii^—Nd2—O5	129.8 (2)
O1^iv^—W2—O6	96.6 (3)	O8—Nd2—O3^x^	139.7 (2)
O1^iv^—W2—O5	106.8 (3)	O7^vii^—Nd2—O3^x^	66.3 (2)
O6—W2—O5	91.3 (3)	O1—Nd2—O3^x^	64.6 (2)
O1^iv^—W2—O3^v^	93.3 (3)	O6—Nd2—O3^x^	125.3 (2)
O6—W2—O3^v^	98.6 (3)	O1^ix^—Nd2—O3^x^	138.9 (2)
O5—W2—O3^v^	156.4 (3)	O2^iii^—Nd2—O3^x^	68.4 (2)
O1^iv^—W2—O9	91.1 (3)	O5—Nd2—O3^x^	64.0 (2)
O6—W2—O9	171.5 (3)	O8—Nd2—O4^iii^	91.3 (2)
O5—W2—O9	83.0 (3)	O7^vii^—Nd2—O4^iii^	134.1 (2)
O3^v^—W2—O9	84.5 (3)	O1—Nd2—O4^iii^	64.5 (2)
O1^iv^—W2—O4^v^	166.5 (3)	O6—Nd2—O4^iii^	133.7 (2)
O6—W2—O4^v^	90.9 (3)	O1^ix^—Nd2—O4^iii^	67.0 (2)
O5—W2—O4^v^	84.0 (3)	O2^iii^—Nd2—O4^iii^	60.1 (2)
O3^v^—W2—O4^v^	74.5 (3)	O5—Nd2—O4^iii^	138.4 (2)
O9—W2—O4^v^	82.2 (3)	O3^x^—Nd2—O4^iii^	95.7 (2)
O1^iv^—W2—Nd2^iv^	44.5 (2)	O8—Nd2—W2^xi^	160.01 (19)
O6—W2—Nd2^iv^	109.4 (2)	O7^vii^—Nd2—W2^xi^	100.57 (16)
O5—W2—Nd2^iv^	144.8 (2)	O1—Nd2—W2^xi^	30.95 (15)
O3^v^—W2—Nd2^iv^	50.0 (2)	O6—Nd2—W2^xi^	128.07 (16)
O9—W2—Nd2^iv^	78.7 (2)	O1^ix^—Nd2—W2^xi^	104.97 (16)
O4^v^—W2—Nd2^iv^	122.34 (18)	O2^iii^—Nd2—W2^xi^	79.85 (16)
O1^iv^—W2—Nd2	120.5 (2)	O5—Nd2—W2^xi^	70.14 (16)
O6—W2—Nd2	45.2 (2)	O3^x^—Nd2—W2^xi^	34.37 (14)
O5—W2—Nd2	49.4 (2)	O4^iii^—Nd2—W2^xi^	73.54 (16)
O3^v^—W2—Nd2	129.1 (2)	O8—Nd2—W2	103.17 (19)
O9—W2—Nd2	127.1 (2)	O7^vii^—Nd2—W2	86.45 (16)
O4^v^—W2—Nd2	72.57 (19)	O1—Nd2—W2	86.55 (17)
Nd2^iv^—W2—Nd2	153.578 (17)	O6—Nd2—W2	31.75 (16)
O5^vi^—Nd1—O9^iv^	108.0 (2)	O1^ix^—Nd2—W2	77.98 (17)
O5^vi^—Nd1—O2^vii^	156.0 (2)	O2^iii^—Nd2—W2	159.08 (15)
O9^iv^—Nd1—O2^vii^	92.4 (2)	O5—Nd2—W2	33.16 (14)
O5^vi^—Nd1—O7	75.9 (2)	O3^x^—Nd2—W2	97.17 (16)
O9^iv^—Nd1—O7	119.6 (2)	O4^iii^—Nd2—W2	139.03 (14)
O2^vii^—Nd1—O7	105.4 (2)	W2^xi^—Nd2—W2	96.814 (16)
O5^vi^—Nd1—O6	79.4 (2)	O8—Nd2—W1^iii^	93.93 (19)
O9^iv^—Nd1—O6	119.7 (2)	O7^vii^—Nd2—W1^iii^	102.97 (16)
O2^vii^—Nd1—O6	79.5 (2)	O1—Nd2—W1^iii^	75.32 (17)
O7—Nd1—O6	120.2 (2)	O6—Nd2—W1^iii^	161.63 (17)
O5^vi^—Nd1—O7^ii^	127.1 (2)	O1^ix^—Nd2—W1^iii^	98.99 (16)
O9^iv^—Nd1—O7^ii^	60.9 (2)	O2^iii^—Nd2—W1^iii^	30.07 (15)
O2^vii^—Nd1—O7^ii^	73.7 (2)	O5—Nd2—W1^iii^	134.69 (15)
O7—Nd1—O7^ii^	69.7 (2)	O3^x^—Nd2—W1^iii^	73.07 (16)
O6—Nd1—O7^ii^	153.1 (2)	O4^iii^—Nd2—W1^iii^	32.17 (14)
O5^vi^—Nd1—O8	90.9 (2)	W2^xi^—Nd2—W1^iii^	66.284 (15)
O9^iv^—Nd1—O8	160.7 (2)	W2—Nd2—W1^iii^	161.709 (19)
O2^vii^—Nd1—O8	70.3 (2)	W2^xi^—O1—Nd2	104.6 (3)
O7—Nd1—O8	60.1 (2)	W2^xi^—O1—Nd2^ix^	148.7 (4)
O6—Nd1—O8	66.9 (2)	Nd2—O1—Nd2^ix^	105.7 (2)
O7^ii^—Nd1—O8	104.5 (2)	W1—O2—Nd1^vi^	145.5 (4)
O5^vi^—Nd1—O3^ii^	66.4 (2)	W1—O2—Nd2^iii^	104.6 (3)
O9^iv^—Nd1—O3^ii^	63.2 (2)	Nd1^vi^—O2—Nd2^iii^	104.7 (3)
O2^vii^—Nd1—O3^ii^	136.4 (2)	W2^xii^—O3—W1	103.7 (3)
O7—Nd1—O3^ii^	64.7 (2)	W2^xii^—O3—Nd2^viii^	95.7 (2)
O6—Nd1—O3^ii^	143.4 (2)	W1—O3—Nd2^viii^	152.7 (3)
O7^ii^—Nd1—O3^ii^	63.0 (2)	W2^xii^—O3—Nd1^ii^	133.4 (3)
O8—Nd1—O3^ii^	123.9 (2)	W1—O3—Nd1^ii^	88.3 (2)
O5^vi^—Nd1—W1^ii^	105.15 (17)	Nd2^viii^—O3—Nd1^ii^	92.2 (2)
O9^iv^—Nd1—W1^ii^	35.29 (17)	W1—O4—W2^xii^	106.1 (3)
O2^vii^—Nd1—W1^ii^	98.77 (17)	W1—O4—Nd2^iii^	95.9 (3)
O7—Nd1—W1^ii^	84.57 (15)	W2^xii^—O4—Nd2^iii^	147.0 (3)
O6—Nd1—W1^ii^	154.94 (18)	W2—O5—Nd1^vii^	130.8 (4)
O7^ii^—Nd1—W1^ii^	34.47 (14)	W2—O5—Nd2	97.5 (3)
O8—Nd1—W1^ii^	136.43 (15)	Nd1^vii^—O5—Nd2	98.8 (3)
O3^ii^—Nd1—W1^ii^	40.51 (15)	W2—O6—Nd2	103.0 (3)
O5^vi^—Nd1—W1	90.51 (18)	W2—O6—Nd1	145.6 (4)
O9^iv^—Nd1—W1	141.30 (17)	Nd2—O6—Nd1	110.3 (3)
O2^vii^—Nd1—W1	80.66 (17)	W1—O7—Nd2^vi^	130.6 (3)
O7—Nd1—W1	31.71 (14)	W1—O7—Nd1	106.5 (3)
O6—Nd1—W1	96.60 (18)	Nd2^vi^—O7—Nd1	102.8 (2)
O7^ii^—Nd1—W1	80.69 (15)	W1—O7—Nd1^ii^	98.3 (3)
O8—Nd1—W1	30.35 (15)	Nd2^vi^—O7—Nd1^ii^	107.6 (2)
O3^ii^—Nd1—W1	96.28 (15)	Nd1—O7—Nd1^ii^	110.3 (2)
W1^ii^—Nd1—W1	107.859 (18)	W1—O8—Nd2	143.0 (4)
O5^vi^—Nd1—Nd2^vi^	42.80 (18)	W1—O8—Nd1	103.6 (3)
O9^iv^—Nd1—Nd2^vi^	106.32 (16)	Nd2—O8—Nd1	110.9 (3)
O2^vii^—Nd1—Nd2^vi^	143.23 (18)	W1^xiii^—O9—W2	138.0 (4)
O7—Nd1—Nd2^vi^	37.87 (14)	W1^xiii^—O9—Nd1^xi^	100.3 (3)
O6—Nd1—Nd2^vi^	115.25 (18)	W2—O9—Nd1^xi^	120.4 (3)
O7^ii^—Nd1—Nd2^vi^	87.96 (15)		

Symmetry codes: (i) *x*+1, −*y*+3/2, *z*−1/2; (ii) −*x*+1, −*y*+2, −*z*; (iii) −*x*+1, −*y*+1, −*z*; (iv) −*x*, *y*+1/2, −*z*+1/2; (v) *x*−1, *y*, *z*; (vi) *x*, −*y*+3/2, *z*−1/2; (vii) *x*, −*y*+3/2, *z*+1/2; (viii) −*x*+1, *y*+1/2, −*z*+1/2; (ix) −*x*, −*y*+1, −*z*; (x) −*x*+1, *y*−1/2, −*z*+1/2; (xi) −*x*, *y*−1/2, −*z*+1/2; (xii) *x*+1, *y*, *z*; (xiii) *x*−1, −*y*+3/2, *z*+1/2.
